# Smart Roads for Autonomous Accident Detection and Warnings

**DOI:** 10.3390/s22062077

**Published:** 2022-03-08

**Authors:** Abdul Mateen, Muhammad Zahid Hanif, Narayan Khatri, Sihyung Lee, Seung Yeob Nam

**Affiliations:** 1 Information and Communication Engineering Department, Yeungnam University, Gyeongsan 38541, Korea; abdulmateen@fuuastisb.edu.pk (A.M.); narayankhatrig@ynu.ac.kr (N.K.); 2 Department of Computer Science, Federal Urdu University of Arts, Science & Technology, Islamabad 45570, Pakistan; muhammadzahidhanif1983@gmail.com; 3 School of Computer Science and Engineering, Kyungpook National University, Daegu 41566, Korea; sihyunglee@knu.ac.kr

**Keywords:** vehicle, road, accident, autonomous, smart road

## Abstract

An increasing number of vehicles on the roads increases the risk of accidents. In bad weather (e.g., heavy rainfall, strong winds, storms, and fog), this risk almost doubles due to bad visibility as well as road conditions. If an accident happens, especially in bad weather, it is important to inform approaching vehicles about it. Otherwise, there might be another accident, i.e., a multiple-vehicle collision (MVC). If the Emergency Operations Center (EOC) is not informed in a timely fashion about the incident, fatalities might increase because they do not receive immediate first aid. Detecting humans or animals would undoubtedly provide us with a better answer for reducing human fatalities in traffic accidents. In this research, an accident alert light and sound (AALS) system is proposed for auto accident detection and alerts with all types of vehicles. No changes are required in non-equipped vehicles (nEVs) and EVs because the system is installed on the roadside. The idea behind this research is to make smart roads (SRs) instead of equipping each vehicle with a separate system. Wireless communication is needed only when an accident is detected. This study is based on different sensors that are used to build SRs to detect accidents. Pre-saved locations are used to reduce the time needed to find the accident’s location without the help of a global positioning system (GPS). Additionally, the proposed framework for the AALS also reduces the risk of MVCs.

## 1. Introduction

Traveling is one of the basic needs of every person who lives in cities or villages. There are several ways to travel from one place to another by air, water, rail, and road in various types of vehicle, e.g., cars, motorbikes, buses, and trucks. Roads are the foremost source of linking between cities and villages. Due to the ease in traveling by road, vehicles have become the main way people travel. The chances of vehicular accidents (Vas) have increased with the growing number of vehicles on the roads. During a journey, one does not know what will happen on the next road, particularly during bad weather conditions (BWC). In such a situation, driving can be difficult due to bad visibility, which can lead to an accident. It was also noticed that in BWC, multiple vehicle collisions (MVCs) can occur owing to delays in receiving information about an incident. According to one study by the Islamabad police, there were 9582 accidents from 2016 to 2017 all over Pakistan, involving 11,317 vehicles, leading to 5047 fatalities and 12,696 persons injured [1].

Vehicles can be divided into two main groups: equipped vehicles (Evs) and non-equipped vehicles (nEVs). Evs have sensing capabilities to avoid or detect accidents. Evs include vehicles equipped with a smartphone-based application or a microcontroller with different sensors. It uses GSM, LTE, or 5G to send messages; a GPS for finding locations; and GPRS, LTE, or 5G for internet connectivity. All old vehicles with no capability for sensing an accident are nEVs. Thus, the benefits from accident detection and alerts are not provided in nEVs. A question arises about why we rely on vehicle sensors or smartphone-based systems. The GSM signal is weak in many distant areas, and communication links might be unstable in those areas. On the other hand, a GPS requires 10 to 15 min to fix a location for the first time, which leads to late broadcasts. The main goal of this research is to:

1. introduce a new framework of smart road based on multiple sensors to save the lives of people injured in an accident, and protect people and vehicles against MVCs;
2. detect Vas autonomously without using vehicular sensors;
3. alert drivers of approaching vehicles about an accident, even without vehicular communications;
4. inform an Emergency Operations Center (EOC) about an accident and its location without needing a GPS.

According to one dictionary, the word accident is defined as “an uncertain event which may lead to injury, loss of life, or property damage” [2]. An accident is also defined as “an event in which one or more vehicles hit in such a way that may lead to loss of life or injury or vehicle damage, resulting in a traffic blockage on the road”. The risks for loss of life, injuries, and other damage may increase if an incident is not reported to an EOC in a timely fashion. Lives can be saved by sending timely information about an accident through an automated mechanism. Moreover, quick automobile accident detection and an alert system are required to protect approaching vehicles against an MVC. Several methods have been implemented in advanced vehicles (Avs) for avoiding an accident. An accident threat is detected through sensors installed in vehicles or by using smartphone sensors. Previous researchers have used accelerometers, smoke detectors, infrared (IR) obstacle sensors, proximity sensors, and biosensors to detect an accident. A microcontroller board collects sensor information. A GPS receiver is used for location tracking and monitoring speeds, while GSM, LTE, and 5G technologies send information about an accident. Event information can include the location and time, videos, and images of the event for the EOC. There are two methods to detect accidents autonomously; we can equip all vehicles, or we can develop smart roads that can sense any type of accident and convey messages wirelessly to an EOC. To protect vehicles around the accident area, a new framework is proposed, i.e., an accident alert light and sound (AALS) system. In this framework, drivers of oncoming vehicles are informed by blinking lights and a siren after detection of an accident. This blinking light is so bright that it can be seen easily in BWC. Wireless communications on these smart roads (SRs) is provided with the HC-12 serial communication module of the Arduino Uno board. These SRs act as live roads because of their sensing power.

The communication protocols can be GSM or 5G technology in vehicle-to-everything (V2X) systems [3] to send messages about an accident. V2X can be realized from integration of cellular 5G and new radio (NR) access technology (i.e., 5G NR V2X). Mobile-phone-based applications use built-in sensor data to detect accidents. One of the major drawbacks of using mobile phone sensors for accident detection is false positives, i.e., incorrectly detecting an accident. The sensors used in mobile phones and Avs need calibration, which is difficult to achieve or may not be possible. When a car jerks due to an accident, mobile technology might turn off, or a battery might be displaced, preventing information about the accident from reaching an EOC in a timely manner.

In this research, an AALS system is proposed to detect automobile accidents and send alerts to other vehicles. No changes are required to nEVs and Evs, because the whole system is installed on the roadside, leading to the new concept of smart roads. A smart road has the capability to detect an incident on the road and send this information to the EOC. In case of an accident, the AALS system uses the event-driven wireless sensor network (EDWSN) protocol [4] for communication between nodes. Otherwise, all the nodes are isolated from each other. Each node works independently to detect an accident. When an accident happens on part of an SR, the AALS system detects it through its sensing capabilities, and generates light and sound alerts. The purpose of these alerts is to inform drivers of approaching vehicles about the accident so they can take precautionary measures, e.g., reduce speed or stop to avoid the accident. In this way, the risk of MVCs is reduced, leading to fewer fatalities and less vehicle damage. With the help of wireless communications, an accident event alert message is sent to a nearby node in a chain process using the EDWSN protocol until it reaches the EOC. The communication takes less than a minute or two to reach the EOC from the node detecting the accident. The message received by the EOC contains all the important information about the location of the node detecting the accident with the complete address, direction of the road, and a fire alert (if fire is also detected). The EOC sends a rescue team with an ambulance and fire brigade (whatever is required) to the accident’s location to help save lives. The AALS system cannot reset itself; it is reset by the EOC rescue team after operations are performed and the road is clear. To reset the AALS system, a hard-reset button is pressed on the node detecting the accident. Then, the reset message is generated and sent to other nodes in a chain process until it reaches the EOC. After forwarding the message, each node resets itself and returns to the normal working state.

Main contributions of this research are:

1. A brief survey on the state of the art related to pre-accident as well as post-accident models, frameworks, and techniques;
2. Identification and reporting of limitations in previous studies related to accident detection;
3. The concept of a smart road with an event-sensing capability, plus implementation and testing through various experiments;
4. Demonstration of a new and modern way to quickly detect accidents and communicate with nearby vehicles and EOCs.

The remainder of the paper is arranged as follows. Section 2 describes previous work related to the detection of accidents and MVCs on roads. It discusses previous studies and highlights their limitations. Section 3 discusses the problem background, and provides insight into the proposed AALS and its main components. Section 4 outlines details on the hardware and experimental setup for the AALS. Section 5 presents the experiments conducted and their outcomes (with graphs). Finally, Section 6 concludes the paper and suggests future research directions.

## 2. Related Works

A large amount of research is being carried out in the domain of accident avoidance and accident alarms by a large number of researchers and practitioners. In order to avoid accidents, many approaches are utilized to enhance safety. For ease of reference, the literature on accident detection and avoidance is separated into three approaches: stand-alone, cooperative, and hybrid. Stand-alone approaches use sensors, such as radar and light detection and ranging (LiDAR), for accident avoidance and detection, whereas cooperative approaches rely on V2X technology, and hybrid approaches combine the two methods.

### 2.1. Stand-Alone Approaches

A system based on a programmable integrated circuit (PIC) microcontroller and an ultrasonic sensor installed in vehicles was proposed by Govindarajulu and Ezhumalai [5]. The ultrasonic sensor’s role is to detect any obstruction, speed bump, steep curve, or humans on the road. The sensor’s input is processed by the microcontroller, which acts as a computing device. If an abnormality is discovered, vehicle drivers are notified so they can take appropriate action, such as slowing down or changing lanes. The time delay is controlled by the microprocessor, and ultrasonic sensors measure the distance by using an echo produced by an impediment to a high-frequency sound pulse. A voice message is created if the distance between the vehicle and the obstacle is less than a preset threshold.

Bahgat et al. [6] proposed model-based visual sensing for avoidance of vehicular accidents. Image processing is implemented for detection of a nearby vehicle to maintain a safe distance by alerting the driver. In their work, a backend server monitors the behavior of various advanced vehicles through sensors and mobile communications. All the calculations and estimations are performed on the server. When the server finds any chance of a collision, it provides alerts to drivers. The proposed system is complicated and involves high computational costs.

Rapp et al. proposed a technique that utilizes a short-range radar for self-driving vehicles by using normal distribution transform (NDT) [7]. The radar is used to predict lane changes with simultaneous localization and mapping (SLAM) and landmark extraction. A semi-Markov process is adopted to model the varying conditions and environments in a 2D representation. The main contribution of their work is a grid map representation where every cell’s dynamics are modeled as a dynamic process. However, their proposed idea can solve the problem of localization only in a limited area.

Barjenbruch et al. [8] estimated the velocity and yaw rate (angular velocity during rotation) with a spatial-data radar sensor. They optimized the discrepancy in position from radar detection and Doppler velocity (to calculate the expected Doppler shift for existing pose hypothesis to each mapped landmark) for w.r.t. moving vehicles. The implementation of this approach in real systems is difficult due to high computation costs. Moreover, even small errors in methods that calculate relative motion between consecutive sensor readings will accumulate when summed over a longer time [9].

In order to drive safely, Hyung et al. [10] used cruise control technology in self-driving automobiles. In the cruise control system, the vehicle can detect and maintain a safe distance from the car ahead of it. Brakes and acceleration are controlled by using the proportional-integral-derivative (PID) approach, which calculates three basic coefficients to produce optimal responses. The PID approach also adjusts the accelerator and brake pedals to maintain a safe distance between vehicles. Distance errors caused by a preceding vehicle can be determined from distance information obtained by a laser scanner installed on the front of a vehicle. Acceleration control in each successive vehicle provides velocity control by transmitting acceleration position sensor (APS) signals, which can be artificially generated by the speed control unit to the electronic control unit (ECU). Their work was tested using simulators (Carsim and Simulink), and proved highly useful.

An automated deep learning (DL)-based system [11] was developed for detecting accidents from video data. The system uses visual components in temporal order to represent traffic collisions. As a result, the model architecture is composed of a visual-features-extraction phase followed by transient pattern identification. Convolution and recurrent layers are used in the training phase to learn visual and temporal features. In public traffic accident datasets, accuracy of 98% was attained in detection of accidents, demonstrating a strong capacity for detection independent of the road structure. The solution is limited to automobile crashes, not motorbikes, bicycles, and pedestrians. Furthermore, the model makes mistakes when determining accident segments under poor illumination (e.g., at night), at low resolutions, and when there are occlusions.

A system suggested by Kim et al. [12] includes a LiDAR sensor to measure speed, and a prediction algorithm based on the stop sight distance (SSD) formula to calculate braking distance. Red light running (RLR) is predicted by a sensor that monitors the speed of the vehicle approaching an intersection. The results demonstrated that RLR can be predicted from the front and side of a moving vehicle using LiDAR and the SSD algorithm. The vehicle’s weight is fixed in this method; however, in practice, the braking distance varies depending on the vehicle’s weight.

Xie et al. [13] suggested a method for recognizing and tracking obstacles in 3D LiDAR point clouds. The point clouds generated by 3D LiDAR after road segmentation are first rasterized, and then additional relevant cells are added. A clustering technique is then used to group the obstacle cells in the next stage. Static obstacles are discovered using a multi-frame fusion approach by evaluating the clusters. Finally, moving obstacles are tracked using an upgraded dynamic tracking point model and a regular Kalman filter. The proposed method was tested in a variety of demanding settings, but has yet to deal with scenarios where barriers are extremely near one another.

A systematic approach to multi-LiDAR data processing [14] was introduced, consisting of calibration, filtering, clustering, and classification. Within the multi-LiDAR architecture, the accuracy of obstacle detection is enhanced by using noise filtering and clustering. The applied filtering method is based on sampling point occupancy rates (Ors). An adaptive search (AS) approach is used to improve density-based spatial clustering of applications with noise (DBSCAN). Furthermore, when AS-DBSCAN is combined with the proposed OR-based filtering, more robust and precise obstacle detection is achieved. The agglomerative hierarchical clustering technique with complete linkage is applied. The same procedure is then employed with a single linkage. If the two targets occupy neighboring segments or share the same segment, and their longitudinal separation is less than 0.8 m, the clustering algorithm utilized might aggregate detection of vehicles into a cluster, resulting in a merged/unresolved report. An inside perception test and an on-road test were performed on a fully instrumented, autonomous hybrid electric automobile. Experiments proved that the proposed algorithms are reliable and practical.

Using LiDAR, a mechanism for estimating the contour of approaching vehicles in the pre-crash phase was explored [15]. The data are initially combined, and an environmental model containing the identified items and their properties, such as position and shape, is computed. The mechanism extracts the main parameters of the vehicles from a LiDAR 3D point cloud through a convex hull algorithm. The relevant vehicle contour and longitudinal axis are then calculated. On both static and dynamic measures, tests on vehicles revealed good accuracy and durability of the estimation. Despite the fact that a convex hull algorithm examines a set of points from the reflection point clusters’ outermost region, it defines its bounds by connecting two neighboring points with straight lines. The accuracy of contour prediction may be affected by the fact that a vehicle’s contour is often a higher degree curve. Furthermore, due to noise-induced change in reflection points, evaluating individual points for analysis can introduce mistakes [16].

### 2.2. Cooperative Approaches

Jerry et al. [17] presented a vehicle-to-vehicle (V2V) and vehicle-to-infrastructure (V2I) communication system that leverages short-range communication technologies. The on-board unit (OBU) and the roadside unit (RSU) are the two primary components of this system. The OBD includes a processor, vehicle sensors, a touch screen, speakers, radio link modules, Bluetooth, and a GPS module. The OBU is mounted on vehicles and connected with the system and the in-vehicle network. The primary function of the OBU is to offer vehicle-to-vehicle communication and to gather diagnostic messages. The RSU is a stationary unit installed on roadways and in other conspicuous locations, such as gas stations and toll plazas, to facilitate V2I communication. The RSU receives and sends information to the OBU for ITS and internet services. Messages about collisions or speed limits are shown on a display screen.

A survey by Eskandarian et al. [18] discussed the latest techniques and algorithms in connected and automated vehicles (CAVs) for the identification, perception, planning, and control of vehicles. They addressed remarkable issues related to decision making on path tracking and control of vehicle communications in a cooperative environment. They mentioned how, in a CAV, stability and loss of signals is the major problem in wireless communications. Finally, issues and challenges unsolved in CAVs were outlined for researchers and vendors.

A framework for safe lane changing in connected and automated vehicles was proposed by Zheng et al. [19]. Their lane-changing approach was based on V2V and V2I communications for better movement and traffic operations. The study suggests that changing lanes is risky when the vehicle’s declaration is more than the threshold value of a leading vehicle. Total time, surrogate safety measures, and traffic waves are among the characteristics used to make lane-changing decisions. A heuristic technique is used to identify the lane-changing zones before simulation. A cooperative strategy is implemented near highway off-ramps via vehicle-based control of the aforementioned applications. The main limitation of this work is that it did not consider the environment of human-driven vehicles and the diversity in calculating lane-changing behaviors in different and unexplored places.

A cooperative neighboring vehicle positioning system (CNVPS) [20] was introduced by Nam et al. that leverages GPS technology and other sensors to improve localization. The GPS is used to provide locations owing to its availability 24 h a day, 7 days a week, and in any weather. The technology uses a local map to identify and interact with nearby cars (using V2V communication) in order to obtain an accurate relative position. It uses maximum likelihood estimation to obtain the correct position of a nearby vehicle. The suggested that CNVPS refreshes positions faster; however, it has a high deployment cost and can only be used for already predicted behavior. To overcome GPS refinement concerns, a DL-based cooperative vehicle localization algorithm, called a graph convolution network-CNVPS (GCN-CNVPS), was proposed [21]. To facilitate V2V communication, the technique uses GPS technology to obtain the coordinates, relative distances, angles to neighboring vehicles, and received signal strength indication (RSSI) information from a basic safety message (BSM). Three DL-based approaches that require less hardware were presented to address the challenge of localization. Finally, comparison results showed that the GCN-CNVPS effectively utilizes temporal and spatial correlations with lower overhead.

Through fuzzy logic and the Dempster–Shafer Theory, a predictive multi-criteria technique [22] was employed for multi-criteria decision-making in Avs following other vehicles and for lane changes. Dempster’s rule is used to identify risk values for hypotheses and trajectories, while fuzzy logic is used to handle uncertain situations and nonlinear data. All calculations are based on parameters such as accessibility, traffic rules, passenger preferences (for comfort), safety, and energy consumption. The fuzzy rules are based on data learning in real time and on traffic rules. The impact of sensors and their relative relevance to criteria and categories, ways to relax if it is safer, and driver preferences are considered for a more flexible decision. This method is scalable and not restricted to the circumstances presented. Using a collision avoidance algorithm (CAA) and a collision avoidance strategy, the authors suggested an enhanced collision avoidance (eCA) technique [23]. The eCA technique is responsible for identifying vehicles’ trajectories in order to avoid crashes. The next vehicles’ trajectories are anticipated in particular by projecting the positions of previous vehicles on curved or straight segments through status indicated by blinking lights. The CAS, on the other hand, regulates the speed of the vehicle in order to avoid accidents. A simulation was run on SUMO and NS-3, demonstrating the effectiveness of the strategy by avoiding nearly every collision under all of the tested circumstances.

Ijjina et al. proposed an object-tracking-based algorithm approach to detect accidents (termed Mask-RCNN) [24]. The chance of an accident is computed using the speed and trajectory of the vehicle. On CCTV surveillance footage, it has a high detection rate and a low false alarm rate. The model was tested in a variety of situations (including rain, storms, and low light), and proved effective. They also noted a low false alarm rate of 0.53%.

An accident management system was proposed in [25] that makes use of cellular technology in public transportation. This method enables communication across various components, including those in ambulances, RSUs, and servers. Furthermore, in this system, an optimal route-planning algorithm (ORPA) is proposed to optimize aggregate spatial utilization of road networks while lowering the travel cost to operate a vehicle. The ORPA was evaluated through simulations, and findings were compared with other current algorithms. In congested areas, the proposed method can also be used to offer fast routes for ambulances. All vehicles, including ambulances, are required to have a route indicator installed, as well as the ability to use remote correspondence. The ORPA outperformed in terms of average speed and travel duration, according to the evaluation data. The proposed system only works for predicted patterns and can fail due to unpredicted behavior of traffic.

With the advancement of 5G technology, V2X, which emphasizes safe driving and traffic management for slightly advanced cars, is rapidly progressing. To ensure the sum rate and compliance with reliability constraints, a unicast communication paradigm [26] was suggested. In a highway scenario, the authors chose signal power and buffer size as the key restrictions for V2X communication. The focus of the study was device-to-device (D2D) resource distribution. Their proposed resource allocation algorithm was applied to resource sharing between user equipment and cellular user equipment. When compared to other existing methods, simulation results revealed that the proposed algorithm had very promising performance.

Another study compared DSRC and LTE in infrastructure mode (LTE-I) and LTE-D2D in terms of average end-to-end delay and packet delivery ratio (PDR) under various communication conditions [27], which were achieved by varying the communication perimeter, message generation frequency, and road traffic intensity.

In the context of V2V, Karoui et al. [28] measured the signal-to-interference-plus-noise ratio and reference signal received power in two scenarios to benchmark ITS-G5 and LTE-V2X (mode 3), in terms of the end-to-end latency and radio frequency conditions. The study looked at the influence of substantial data traffic on an ITS alert service, and the influence of handover on ITS safety services in the context of V2V.

Along with MEC, blockchain can be used to protect and manage 5G V2X. Other research revealed the current condition of V2X, as well as its evolution based on cellular 5G and non-cellular 802.11bd [29], exploring how blockchain can be integrated with 5G-based MEC vehicular networks. With future research initiatives, the concerns and limitations in existing edge computing and 5G V2X are addressed.

### 2.3. Hybrid Approaches

Adithya et al. [30] created a method for vehicular communications using ZigBee wireless technology in order to prevent accidents. A GPS modem is utilized in this system to determine an accident’s exact location. Blynk applications on smartphones are used to save data to the cloud. When a car’s distance from a neighboring vehicle is lower than a certain threshold, the vehicle uses ZigBee to send messages to the adjacent vehicle, alerting the driver to take suitable action.

A collision avoidance system was developed [31] based on ZigBee where GPS technology is used to find a vehicle’s current location. In this system, the brakes of the vehicle are controlled using the automatic braking system (ABS) in the recent vehicles. The proposed model is used in Avs with a protective system already installed. The research they carried out was limited to line-of-sight visualization by a camera and IR sensors.

Many researchers and practitioners have adopted cameras, radar, and LiDAR in vehicles with a GPS for safe or autonomous driving. For the study in [32], the localization problem was solved by using radar data (pulse-based short-range radar) of the route in relation to a fresh traversal. The approach requires only the latest traversal history for the route, rather than storing and processing large amounts of data. The proposed approach was tested and validated to localize a truck on a road by using a five-minute dataset. Results after the experiments showed root mean square (RMS) errors of 7.3 cm laterally and 37.7 cm longitudinally, with the worst case being 27.8 cm and 115.1 cm, respectively [33]. Another problem with this approach is unexpected objects in dynamic surroundings.

In recent years, optical camera communication (OCC) [34] has been employed for communication between vehicles and RSUs. For a variety of activities, including traffic sign recognition and determining the distance between two vehicles, OCC employs a light-emitting diode (LED), an image sensor, and infrared (IR) rays. In a vehicle ad hoc network (VANET), the highest data rate reached to date with OCC is 55 Mbps. That paper included a survey of the literature on OCC, receiver and transmitter topologies, as well as some open research problems. Lee [35] investigated a highway accident detection system using CCTV and a Calogero–Moser system (an integrable system used at a crossroads). The effort attempted to overcome the difficulty in object recognition in shade and nighttime environments. In order to develop an accurate detection system, the flow of a vehicle trace was discovered to be similar to a Wigner distribution in terms of level spacing. Later on, Lee developed an advanced road traffic analytics processing system [36] that can process and analyze all the data in near real time. The suggested framework was evaluated using data collected from 41 CCTV cameras along the Icheon-to-Gangneung highway in Korea. The prediction method was implemented in a neighborhood between CCTV cameras.

Wei et al. [37] suggested an approach in which LiDAR and a camera are utilized to detect and prevent a vehicle from entering a restricted space. The beacons were normal orange traffic cones with a vertical luminous pole. They reduced false-positive LiDAR detection by projecting beacons in camera footage using DL, and by validating the detection using neural-network-learned projection from the camera into the LiDAR space. The utility of the suggested approach was proved via data acquired at Mississippi State University’s Center for CAVS.

Al-Mayouf et al. [38] proposed a system for accident management using cellular-technology-based VANETs. This technique ensures real-time communication between various vehicles, including crashed vehicles and ambulances, and roadside communicating units that send all updates to a server. An algorithm is used for optimal routing of an ambulance to its target, i.e., the location of the injury. In a vehicle, two types of sensors are installed: one is a bio-medical sensor that helps monitor the heart rate of the driver, and the other is a vehicular sensor to collect information about acceleration, temperature, tire pressure, etc. The microcontroller obtains inputs from these sensors and manipulates them to find any abnormalities. If abnormal values are found, it obtains the current location through a GPS module. After obtaining location information, it sends the information to nearby RSUs, which forward it to a central server to guide the ambulance to the target location. The drawback of this technique is that if the cellular signals are not present or weak, the required communication may fail, and the delay may be life-threatening.

Sarmin et al. [39] used GSM, a GPS, and an accelerometer-based system for detecting accidents. It provides alerts by sending an SMS to an emergency number when any accident is detected. The Arduino microcontroller is used for processing. The GPS and the accelerometer are attached to input pins, and GSM is used for the I/O port. The processor is responsible for all calculations performed locally, and then an alert is sent to an emergency number using the GSM cellular network. However, the proposed solution has no ability to inform other vehicles about the incident.

Fernandez et al. [40] developed a system to detect accidents using GPS information. In this technique, the receiver extracts the complete GPRMC sentence by demodulating RF signals from GPS satellites. The sentence holds all necessary information about the speed, time, and location of the vehicle. The last two values for each attribute are stored in memory and manipulated by the local processor for detection of any abnormalities. If the processor finds an accident, it raises a HIGH flag that results in autonomous initialization of emergency procedures. Before sending the message, the processor waits for manual cancellation by the driver if no help is required. After five seconds, an SMS alert is generated by the processor with the destination of the emergency.

Ali et al. [41] proposed a smartphone-based accident detection and notification system. The internal sensors of a smartphone are used to detect accidents, and the camera is used for recording images and video. If an accident happens, the video and/or images are sent to an emergency number. One major drawback of this approach is that every smartphone is prone to false positives.

Fernandes et al. [42] built an application for Android phones with an algorithm for autonomous detection of accidents. Mobile phones are connected to a single-board computer (SBC) through a USB serial cable. The accident detection algorithm takes values from the internal sensors of smartphones and vehicles. Whenever an accident occurs, the application detects it and broadcasts a message to all nearby vehicles. The nearby vehicles are informed by the color on the phone screen: green means the road is clear, and red means there is an accident nearby. Every smartphone, however, is prone to false positives.

Dias et al. proposed and developed a system that utilizes GSM, a GPS, the IoT, and GPRS technology with different vehicular sensors and microcontrollers [43]. An Arduino microcontroller receives input from vehicular sensors and sends a message via GSM modules using cellular technology if it obtains an abnormal value. Information about an incident’s location is taken from the GPS. The proposed system is expensive due to the multiple technologies and sensors.

Masini et al. [44] investigates a possible approach toward 5G VAN. The vehicular networks can lease available spectrum from existing cellular networks through base stations (BS) or access points using dynamic spectrum access (AP). They compared LTE performance from current legacy solutions to projected 5G trends. Shrestha et al. [45] investigated an evolution of V2X communication based on 802.11bd and 5G NR, and explain that multi-access edge computing (MEC) can bring cloud closer to vehicular nodes. They also introduce a combination of blockchain and 5G-based MEC vehicular networks as a possible solution for the security, privacy protection, and content caching issues in the existing 5G V2X environment.

The study [46] looks at how well vehicle visible light networks (VVLNs) perform in terms of increasing message delivery rates via full-duplex transmission (FD). According to their tests in which they compared their results with half-duplex (HD), it was discovered that using FD in urban environments increased message delivery ratio by 10%.

By putting sensors on the road, the VANET is integrated with the low-cost wireless sensor network (WSN) to buffer and convey information concerning unsafe situations [47]. The designed system’s prototype has been implemented and tested in the field. The findings show a variety of design tradeoffs and show that right parameters can provide sufficient safety and energy efficiency. In contrast to our work, this study started with the goal of improving driving safety by providing information about road conditions. They gathered and processed sensor data in order to extract information useful for safe driving and transmit it to vehicles that require it.

A novel biologically inspired networking architecture [48] was presented in order to take advantage of the first available DSRC-equipped vehicles as temporary RSUs. Cars acting as temporary RSUs can halt for short periods of time and operate as a communication bridge for other vehicles in the network. Designing local rules and the algorithms that apply them is the basis for the suggested approach. According to results, the proposed method significantly improves message reachability and connection. In order to reduce RSUs, they deployed vehicles as RSUs to improve message reachability and network connectivity.

A reinforcement learning (RL) algorithm [49] is utilized for partially observable intelligent transportation systems (ITS) based on dedicated short-range communications (DSRC). This system’s performance is evaluated for various car flows, detection rates, and road network topologies. This method can effectively reduce the average waiting time of vehicles at a junction even with a poor detection rate. However, their work focuses on reducing traffic congestion by controlling traffic lights, instead of reducing or responding to traffic accidents efficiently.

A deep reinforcement learning-based resource allocation system [50] was developed that includes remote radio head grouping and vehicle clustering. It balances system energy efficiency with service quality and dependability. In terms of performance, noise ratio, feasible data rate, and system energy efficiency, the new method is compared to three current algorithms using simulations. In comparison to our work, this study aims to assign various communication resources in order to improve system capacity while consuming the least amount of energy possible from periodic message traffic overheads.

### 2.4. Analysis of Previous Approaches

All the previous accident-related techniques are based on some sort of continuous monitoring in the vehicle of its surroundings through various sensors with the help of a microcontroller-based processing unit. Calibration of these devices from time to time is necessary for proper function, which becomes costly. Communication between vehicles is carried out by wireless technology. Although a GPS offers easy and accessible localization, the precision of the GPS still has room for further improvement in providing accuracy. To be more specific, a GPS suffers influences from several factors (e.g., receiver noise; a multipath effect), such that the received GPS coordinates have large errors in the actual coordinates of the vehicle, thereby posing a threat to the safety of Avs or the precision of ITS applications [21]. Another problem with GPS technology is that not all driving surfaces have satellite visibility [32]. The received GPS data can be influenced in urban areas by building occlusions, making the data less accurate [13]. On the other hand, post-accident techniques use a GPS to detect and find the location of the accident, with GSM and 5G technology for messaging to emergency service centers [51,52]. These techniques require internet connection. Sometimes, owners do not want to make changes in their vehicles, such as installing vehicular sensors. Another issue is the calibration of these sensors from time to time to avoid false readings. Sensor and communication-link checks for proper function are not always easy. Most of the previous techniques for accident detection apply to Evs only. Our work is also different from other studies [44,45,46,47,48,49,50] where the major focus was to provide information about road conditions to drivers, reduce traffic congestion by controlling traffic lights, and manage communication resources to improve system capacity. A summary on the analysis of previous approaches is provided in Appendix A.

## 3. Proposed Accident Alert Light and Sound System

In BWC, MVCs can happen where a number of approaching vehicles can lead to another accident. In this case, damage and the number of injured people, and/or fatalities might increase. The common cause for this type of accident is poor visibility whereby drivers cannot see the accident until they come upon it (approximately 10 to 15 m away). At that distance, braking will not stop the vehicles in time and, as a result, they become part of the accident. In modern vehicles, some preventive and protective systems are installed for the safety of the driver and passengers. However, in Third World countries, for example, more than 80% of vehicles have no factory-installed protective systems [53]. Until now, no system can handle such situations for all vehicles in an old or late model. Based on the literature review, almost all the work carried out so far is either used for modern vehicles or to make older vehicles into Evs. Evs have some system installed in either a mobile phone application or a microcontroller-based system. All vehicles that have any type of preventive measure and alert system are considered equipped vehicles. Evs act as a source generating accident alert messages. However, if a vehicle is damaged badly or burned in an accident, the system may fail, and no information may be sent to an EOC. In a mobile-phone-based system, the mobile phone may be damaged in an accident. It is not easy to convert all old vehicles into Evs due to financial costs. Another limitation is the need for timely calibration of the sensors in smartphone-based and vehicle-based systems, which is difficult to do. Mobile phones are often prone to false positives, and GSM signals may drop in some areas. If a GPS is used to find locations, major drawbacks are inaccurate and depend on satellite signals. It is difficult to use pre-saved locations while vehicles are moving. It is also notable that no research work has been carried out for accident detection by nEVs.

There is a need for a system that works from outside the vehicle for detection of an accident and generating alerts for approaching vehicles in order to avoid MVCs. A new concept of making smart roads is introduced here. SRs have different types of sensors and actuators installed for autonomous detection of accidents. SRs have nodes (a completely independent system) that are nearly 50 m apart. Because these nodes are fixed on the roadside, it is possible to use their pre-saved locations. A node that detects an accident sends its pre-saved location to an EOC for the quickest rescue operation, saving lives and reducing damage. The main part of the SR is an alert system, called an AALS, which makes drivers of approaching vehicles aware of an accident.

The AALS is a system designed for nEVs and EVs to avoid MVCs. Most of the previous researchers have tried to build a system in which vehicles are able to sense and communicate with each other through devices. In short, they try to make all vehicles equipped. However, equipping all vehicles with the same type of prevention and communication mechanism is difficult to achieve. Modern vehicles may have a factory-built protection system installed. In the system proposed here, a golden yellow blinking light is used with a siren to alert vehicles to an accident. According to one study of different colored lights, red and golden yellow light are visible from a long distance and in bad weather [54]. Red lights are already used for traffic signals; thus, using golden yellow light is a good option. Another parallel way of alerting drivers is with a loud siren, so drivers can hear it and take precautionary measures to avoid being involved in an MVC, especially in BWC. These alerts are generated through an auto mechanism. The proposed system will be installed on both sides of the road. A complete system based on applied studies was built with hardware (including microcontroller boards), different sensing units, and with alert functions. A node can be powered from existing light poles on the roads or highways; otherwise, solar panels can be deployed.

**Figure 1:**
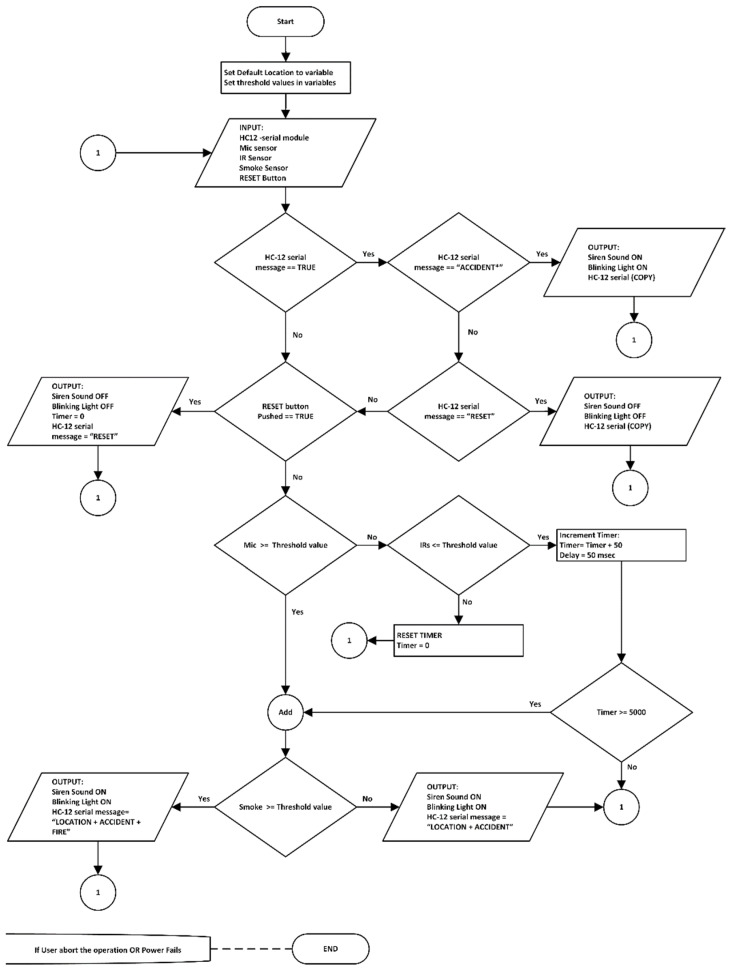
Proposed algorithm for an AALS node.

The basic functions of the AALS system are accident detection from outside vehicles (i.e., from the roadside) and alerting oncoming vehicles about an accident by blinking a light and making a sound. The AALS system communicates with the next node through EDWSN, a wireless communication protocol that uses two HC-12 modules with low power consumption. It functions only when an accident is detected by the AALS or when the reset button is pressed. When an accident happens, certain events occur. For example, when brakes are applied at high speed, a squealing sound is produced; when a vehicle hits another vehicle, it produces a loud sound that can be heard from a distance. If glass breaks, it produces an audible sound; if a vehicle is burning, the temperature of the environment rises, and smoke is produced; when a vehicle suddenly stops in the middle of the road, it becomes an obstacle for other vehicles. By considering all these factors, a foolproof system is available for accident detection. The steps of the proposed algorithm for a node in the system are shown in Figure 1.

### A Way to Smart Roads (SRs)

SRs are roads that have some sort of sensing power given with the help of different types of transducers (devices that convert one form of energy to another) as well as control devices with communication capabilities. Several nodes are installed on the sides of the road to enable sensing at those points. By increasing the number of nodes, sensing power can improve. These nodes hold all the necessary sensing devices and a microcontroller board with a wireless communications system. The distance between nodes is directly proportional to the transmission power of the sensing devices. SRs do not discriminate among vehicle types, and can detect accidents involving Evs as well as nEVs. Similarly, SRs generate light and sound alerts that can be seen and heard by drivers and passengers of approaching vehicles.

**Figure 2:**
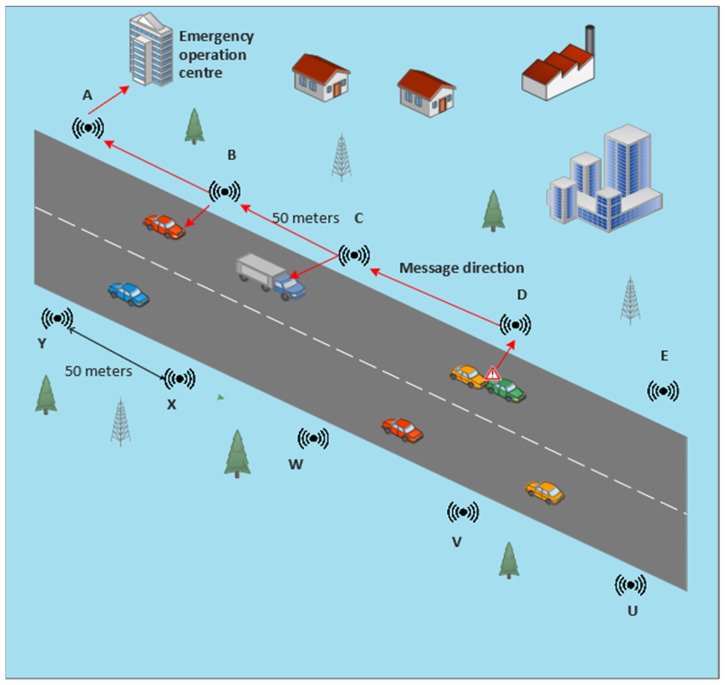
Illustration of a smart road.

A smart road has sensing capabilities for accident and/or event detection. A two-way road is shown in Figure 2, each side with AALS nodes which are installed at a distance of 50 m apart. Labels A, B, C, etc., denote the sensing nodes, and each node communicates with neighboring nodes. If a sensor of node D detects an accident, it sends a message to its immediate downstream node, i.e., node C, which will send the received message to node B, and this procedure is repeated until the message finally reaches the EOC. Similarly, on the other side of the road, if an accident is detected by node Y, it sends a message to node X, which relays the message to its previous immediate node, i.e., node W. This procedure is repeated until the message reaches the EOC. A message cannot be received by any node other than its immediately adjacent node. For example, if node D senses an accident, the message is sent to C, B, A, and the EOC. Different channels are used to perform this type of communication. Each channel is in the 400 kHz bandwidth range. To avoid overlapping signals, the immediately adjacent node should be a part of five channels. The transmitting channel and baud rate of one node are similar to the receiving channel and baud rate of its previous adjacent node. For example, if channel 20 is selected for the transmission of a message for node C, then the same channel (i.e., 20) should be selected for node B and other previous adjacent nodes.

**Figure 3:**
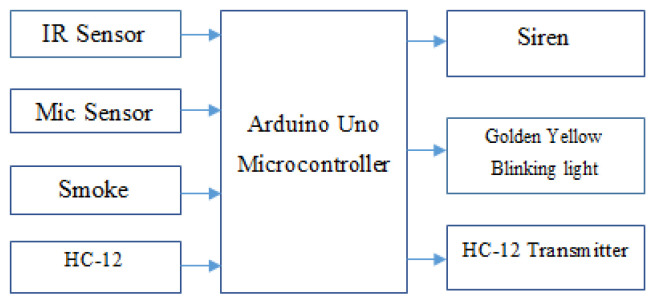
Block diagram of a node in the proposed AALS system.

A functional block diagram of a node in the proposed AALS system is shown in Figure 3.

Approximately 4000 nodes are required to cover a 100-km two-lane road. Each node has the ability to sense and recognize an accident from loud sounds or obstacle detection by using its different sensors. The Arduino Uno microcontroller takes input from the IR, microphone, smoke sensors, and the HC-12 receiver. A smoke sensor is used to detect fire. When a sound is generated due to an accident between vehicles or by a vehicle hitting an object, the microphone picks up the sound and sends it to the microcontroller, which compares the level with a preset threshold. It declares an accident if the level is higher than the threshold. However, if the sound is not greater than the threshold, the microcontroller discards it. In the meantime, the IR sensor works in parallel to detect obstacles on the road. The microcontroller compares the IR value to check whether it is lower than the set threshold and if the elapsed time is more than five seconds (the time taken by a long vehicle to pass in front of the IR sensor at 20 km/h). When an obstacle remains for more than five seconds, the microcontroller recognizes it as an accident. Meanwhile, if the smoke sensor detects any smoke in the atmosphere, the value of the fire bit changes to HIGH. The controller executes three actions if an accident is detected. First, it blinks the light continuously until the system is reset; secondly, it sounds the siren to alert drivers of approaching vehicles so they can take measures to avoid an MVC; and third, it sends a message through the HC-12 transmitter to the immediately adjacent node. A message contains three types of information: the pre-saved location of the detecting node, the direction of traffic on the road, and fire information (If recorded). Communication between nodes is one way, i.e., back toward oncoming vehicles to make their drivers aware of the incident ahead. Every node does the same job in case of accident detection until the message reaches the EOC. When the EOC receives an accident message, it sends an ambulance to the incident location and the fire brigade in case of fire. A rescue operation will be performed to save the lives by giving treatment and hospitalizing the injured peoples if necessary. After the road is cleared by the rescue team, the first communication node will be reset via the hardware push button. Four functions activate when the reset button is pressed: first is to switch off the blinking light; second is to stop the siren; third is to send the RESET message to its adjacent node; and fourth is to reset the controller to its initial state. The RESET message is also sent backwards and will be relayed until received by the EOC. This message indicates to the EOC that the road is now clear, and the rescue operation completed successfully.

## 4. Hardware and Experimental Setup

Developing and testing the entire system on hardware is an interesting part of the proposed work. The experimental setup, microcontroller boards, and sensors are selected, considering the cost and availability. We selected an Arduino Uno R3 board and a PIC 4550 microcontroller [55,56,57] with different sensors and actuators. The Arduino website [57] is an open-source helping website that provides a basic code for different sensor modules that work with Arduino boards. The proposed AALS system is built to minimize computing power needed to detect accidents, and does not need artificial intelligence or a machine learning technique. With the AALS system, accidents are detected by monitoring input from different sensors and comparing them to threshold values. After the successful detection of an accident, the alerts are generated to protect oncoming vehicles from MVCs. The help request message is generated and sent wirelessly to the immediately downstream node. All nodes are interconnected wirelessly in such a way that each node listens to the next node and sends a message downstream to the node. Messages are sent only in case of an accident or after a RESET event, where the RESET button was pressed. To build the whole system, the following sensor modules and components are used:

1. Arduino Uno R3;
2. An IR sensor module
3. A microphone sensor module;
4. A smoke detection module;
5. A GPS 8M module;
6. A HC-12 wireless communication module;
7. Breadboards and jumper wires;
8. A relay module;
9. A golden yellow light and siren;
10. 16X2 LCD with an I2C module.

### 4.1. Arduino Uno R3

**Figure 4:**
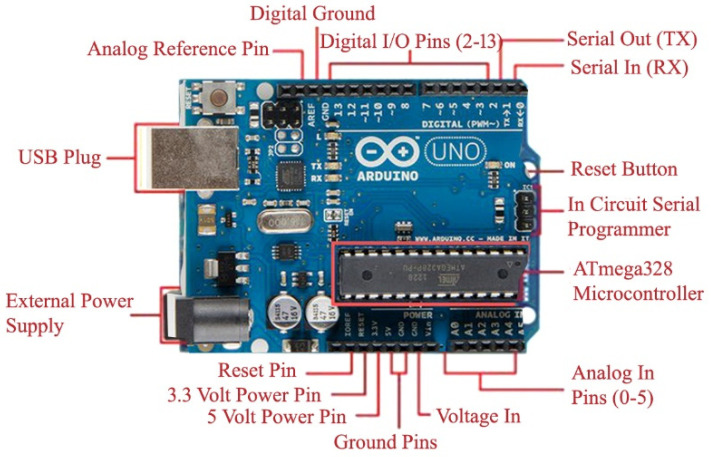
Arduino Uno R3 pin descriptions [55].

An Arduino Uno R3 board is microcontroller-based open-source prototype hardware [57,58] that can be programmed through software called the Arduino IDE. The programming language for the Arduino IDE is C++. Arduino boards have several digital and analog pins, where the digital pin is configurable for input or output by using the “pinMODE” command. Digital pins have only two states: HIGH (1) and LOW (0). However, analog pins have a value that varies from 0 V to 5 V. Most of the sensors, e.g., temperature, sound, and smoke, have analog output, although some provide digital output based on some threshold value set by an installed potentiometer. There are various kinds of Arduino boards available: the Arduino Uno R3, Mega 2560, Due, Ethernet, LilyPad Arduino 328, and their variants. In this research, the Arduino Uno R3 is selected due to its functionality and cost. It works on a 5V DC supply from a USB port. When not connected to a USB port, it can operate on 7 V to 12 V DC from an external adapter. The pins of this board are shown in Figure 4.

### 4.2. IR Sensor Module

**Figure 5:**
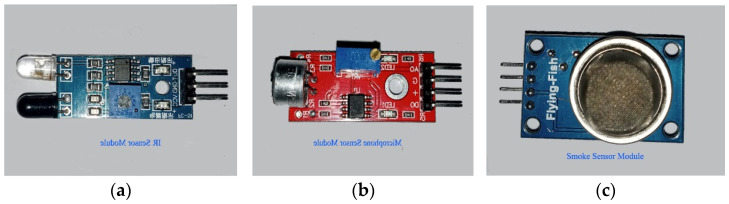
(**a**) IR sensor module [55]; (**b**) microphone sensor module [60]; (**c**) smoke sensor module [61].

This module consists of two parts: the IR transmitter and the IR receiver. The IR transmitter is based on an infrared LED that emits light at an infrared frequency, while the IR receiver is based on a photodiode that can sense reflected infrared light and convert it into an electrical signal [59]. Figure 5a shows the IR sensor module with its three pins. Two pins are used for powering up the module (+5 V and GND), while the third pin is used for output. The output is HIGH when light is not reflected back and vice versa. The range of sensitivity can be adjusted through a variable. This module is normally used for obstacle detection; however, it can be used for counting objects. such as people, vehicles, etc.

### 4.3. Microphone Sensor Module

A microphone is a device that converts sound energy waves into electrical signals. There are two main types of microphone: dynamic and condenser microphones. The microphone sensor module for this system is the condenser type. There is a built-in transistor to amplify the electrical signal generated by the sound wave energy. The strength of the electrical signal is proportional to the loudness of the sound waves, and the frequency is equal to the frequency of the sound waves. There are four pins in this module; two are used for powering up, i.e., +5 V and 0 V, and the other two are output pins. One pin is for analog output, and the other is for digital output. The digital output pin goes to HIGH when the loudness of a sound is greater than the threshold set with a variable; otherwise, it remains at LOW. The voltage of the analog pin varies from 0 to 5 V, depending upon loudness (the energy carried by the sound waves). The analog voltage is measured in numbers from 0 to 1023. Therefore, 0–5 V is divided into 1024 equal-length intervals. The microphone module is shown in Figure 5b.

### 4.4. Smoke Detection Module

The smoke detector is used to detect smoke or fire by sensing combustible gases present in the atmosphere that ionize into a substance present between the two conducting plates. When ionization occurs, varying voltages are produced, indicating harmful gases in the air. The smoke sensor is pictured in Figure 5c. There are four pins in this module: two are used for powering up and the other two are output pins (analog and digital).

### 4.5. GPS 8M Module

**Figure 6:**
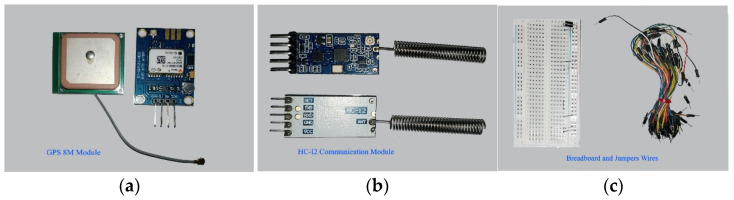
(**a**) GPS 8M module [62]; (**b**) HC-12 module [63,64,65]; (**c**) breadboard and jumper wires [56].

A GPS receiver receives radio frequency (RF) signals at a very high frequency, as shown in Figure 6a. A GPS receives static data broadcast from different geo positioning satellites. These data contain information about the location, time, altitude, etc., in the form of National Marine Electronics Association (NMEA) sentences. These sentences are difficult to understand, and are therefore parsed by the website (www.freenmea.com) (accessed on 21 April 2021). To obtain the latitude and longitude, the parse button is pressed after copying a sentence that starts with $GNGLL to the website. These coordinates are transferred to the Google website to determine the physical location.

### 4.6. HC-12 Wireless Communication Module

The HC-12 is an RF transmitter and receiver used for wireless communications. These modules replace wired communication with a half-duplex wireless serial port. Half duplex means that one module transmits data while another receives it. The HC-12 module is used because of its long range up to 1 km and because its operating frequency ranges from 433 to 437 MHz. Figure 6b shows the pair of HC-12 modules in the proposed system. The frequency range is divided into 100 channels with each having a 400 kHz bandwidth. By selecting different channels, they can communicate with different devices. Modules with the same baud rate and on the same channel can communicate with each other within their communication ranges [62,63,64,65].

### 4.7. Breadboard and Jumper Wires

A breadboard is a device for temporarily making electronic circuits without a permanent connection from soldering. Jumper wires are used to connect different components on the breadboard to control devices, such as Arduino boards. The breadboard consists of five interconnected holes vertically separated from other vertically interconnected holes. The horizontally interconnected holes for the + supply connection are separated into other horizontally interconnected “–” supply connection holes line. A power connection is present on both horizontal sides of a breadboard. There are two types of jumper wire; one is male-to-male and the other is male-to-female. Male-to-male jumper wires connect the breadboard and the Arduino Uno board, whereas male-to-female jumper wires connect different sensors directly to the Arduino Uno board without a breadboard. A breadboard and male-to-male jumper wires are shown in Figure 6c.

### 4.8. Relay Module

**Figure 7:**
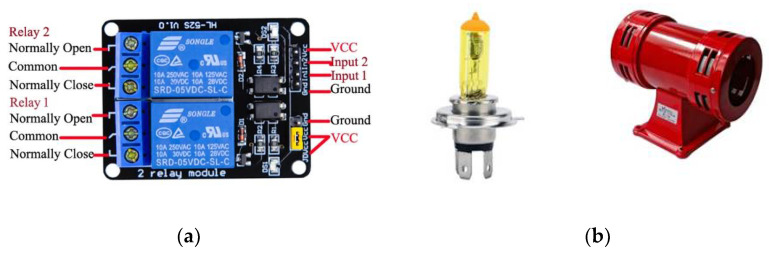
(**a**) Two-channel relay module with pinouts [66]; (**b**) golden yellow light bulb and siren.

A relay module is used with the output of a controlling device that requires more power to operate than its normal output. An Arduino Uno board has a maximum 40 mA output current, which can light up an LED, but cannot control the motor, light bulb, or siren, which operate on high voltage and current rates. Another use of a relay module is to isolate and protect the controller from the output load. A relay module may have one, two, four, eight, and twelve relays for controlling one, two, four, eight, and twelve devices, respectively. Here, two relay modules are used to control two output devices (i.e., the light and siren). Figure 7a shows the two-channel relay modules for the Arduino Uno board.

### 4.9. Golden Yellow Light and Siren

It is difficult to see white light in the daytime or when fog or smoke is in the air. Using a white light does not help people see things in bad weather (e.g., heavy rainfall, storms, or fog). Red lights and golden yellow lights can be seen in such situations from very far away [47]. Red lights are already used in traffic signals. However, so far, golden yellow light is not used in traffic management for any type of alert. Therefore, it is a good choice for an alert signal when it blinks. The bulb that produces golden yellow light to accompany the siren is shown in Figure 7b. The siren is used for alerts by generating a specific loud sound that can be heard from a distance. It is used where a generic alert to the public is required. In the same way, the siren is used in this proposed system to alert drivers of approaching vehicles about an incident ahead.

The last module is the LCD 16×2 which is used to display information at a node during the installation.

### 4.10. Wiring Diagram of an AALS System Node

**Figure 8:**
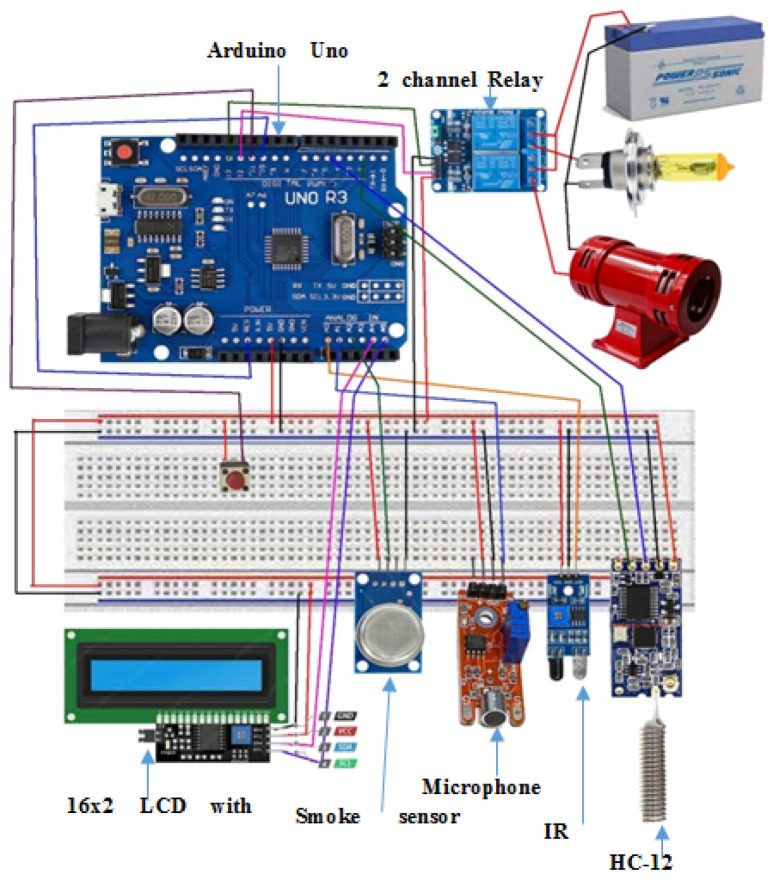
Wiring diagram for the AALS system node.

The pinout connections shown in Figure 8 are used in this research. The HC-12 module has four pins: the VCC pin is connected to +5 V, the GND pin grounds the module, the RX pin connects to pin 5, and the TX pin connects to pin 2 of the Arduino Uno board through a breadboard. LCD 16X2 with the I2C module also has four pins where the VCC pin is connected to +5 V, the GND pin grounds the module, the SDA pin connects to analog pin A4, and the SCL pin connects to analog pin A5 through the breadboard. The IR obstacle module has three pins: the VCC pin connects to +5 V, the GND pin grounds the module, and last output pin is connected to analog input pin A0 through the breadboard. The microphone sensor module has four pins, with the VCC pin connected to +5 V, the GND pin grounding the sensor, digital output not connected to any pin, and the analog output pin connected to A1 of the Arduino Uno board through the breadboard. The smoke sensor module has four pins: the VCC pin is connected to +5 V, the GND pin grounds the module, digital output is not used, and the analog output pin connects to analog input A2 of the Arduino Uno board through the breadboard. The siren and golden yellow light are connected to the Arduino Uno board through a relay module that isolates the output of the Arduino from the high-powered output devices. The two-channel relay module has four input pins: the first is VCC (+5 V) and the second is GND (0 V), with the third and fourth pins being connected to pin 12 for the siren and digital pin 13 for the light, respectively. The relay module controls output devices that operate at 5 V; however, it can control ON or OFF voltages from 5 to 30 V from a DC supply and form 110 to 220 V from an AC supply. It connects a 12 V battery to the siren and light.

In the AALS system, two HC-12 modules are used: one for transmitting messages and the other for receiving messages. The pin connections are such that for the HC-12 transmitter, the Tx pin is not connected, while the Rx pin is not connected for the HC-12 receiver module. This setting is made for one-way communication. If we use only one HC-12 module, then it sends and receives messages on the same channel; hence, the repeated message of the other node is received back in a loop. If a message is received in a loop, it results in a microcontroller being busy, and communication takes place all the time, which is not acceptable. Two HC-12 modules are used to minimize the risk of loopback communications and to ensure only event-driven communications.

The number of sensors deployed on a node is determined by the coverage area and distance. Because the IR sensor has a range of five meters, a node will require 10 IR obstacle sensors. If we replace the IR obstacle sensor with a TF mini IR time-of-flight distance sensor, the number of sensors required drops to four. The smoke sensor can detect smoke at a radius of 7.5 to 10 m (a diameter of 15 to 20 m); hence, three to four smoke sensors are mounted on a node [67]. Microphone sensors have an eight-meter coverage range; hence, six to seven microphone sensors are installed on each node. However, some directional microphones perform at up to 40 m; thus, two directional microphones are used in the proposed system. For a 50-m distance, one siren and one golden yellow light are required.

## 5. Experiments

Different experiments are performed to find the threshold values for the different sensor modules which will be used for the detection of accident. These values have great importance in the successful detection of false-proof accident detection systems.

### 5.1. Finding Threshold for the Microphone Sensor Module

**Figure 9:**
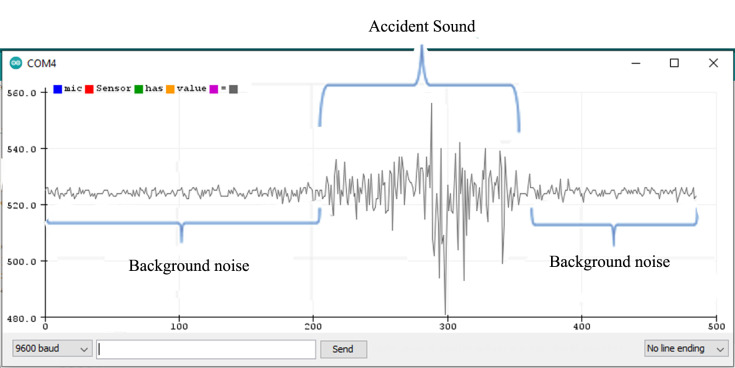
Microphone output on the serial plotter.

The microphone sensor module is attached to the Arduino Uno board, as shown in the wiring diagram from uploading a sketch (a program) to the Arduino board through the Arduino IDE. The sketch contains the basic pin configuration of the Arduino board and the serial print command for showing the incoming values on the serial monitor or serial plotter. The serial monitor is an output screen that shows data on the monitor and the serial plotter shows the output in the form of a graph which can be easily understood. Figure 9 shows the microphone output of the serial plotter.

**Figure 10:**
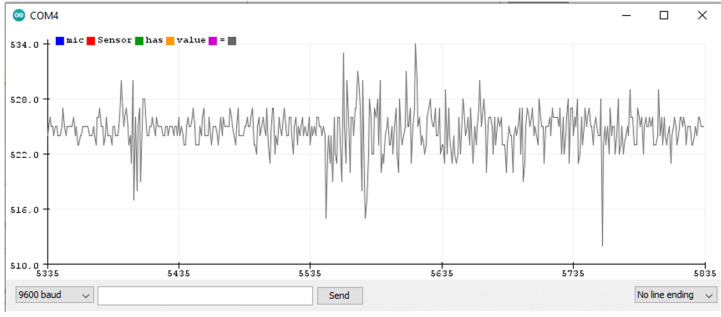
A passing vehicle’s waveform.

**Figure 11:**
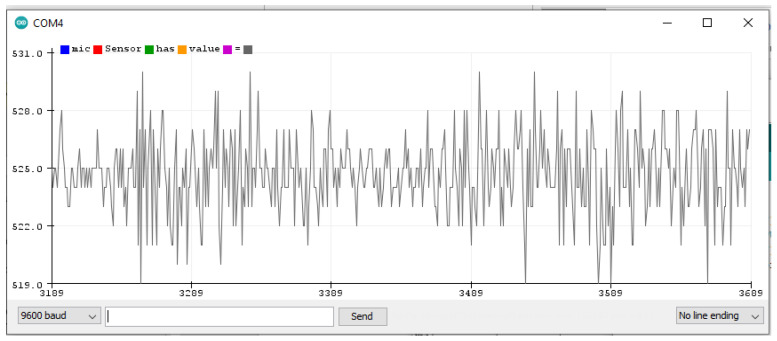
Background noise waveform.

When no sound is produced externally, the microphone senses only background noise. In Figure 9, background noise is shown before and after the accident’s sound. When a sound is played through a loudspeaker, the amplitude increases. The normal background sound ranges from 518 to 532 ADC (calculated by taking the ratio of the analog signal’s voltage to the reference voltage: ADC = (11.003 * dB) − 83.2073 [68]). However, when an accident sound is produced, the range suddenly changes from 480 to 555 ADC. If the range exceeds 532 ADC or is less than 518 ADC, there is a chance of an accident. To make it more precise, another experiment was completed by playing the sound of a passing vehicle into a microphone through a loudspeaker. Figure 10 shows the waveform for the sound of the passing vehicle on the serial plotter. The range of the output waveform varies from 512 to 534 ADC. Figure 10 shows that it varies from 519 to 531 ADC against the background sound. It was noted that there are very small differences in the amplitude of the waveform produced when only background noise is presented or from sound due to vehicles passing by, as shown in Figure 11. In one case, the amplitude of the waveform reached 540 ADC when an accident sound is produced. Various accident sounds are produced, and a similar effect provided loudness that is nearly the same. An accident can be detected from the loudness of sound.

### 5.2. Finding a Threshold for the IR Sensor

The IR sensor module is simpler than the microphone sensor because it produces analog output nearly equal to 5 V; in other words, on the Arduino serial monitor, the value is greater than 1000 when there is no obstacle present. However, the serial monitor showed a reading of less than 20 when an obstacle came in front of it. An obstacle in the module’s light passageway is easy to detect. However, the problem is that it detects every obstacle that passes in front of it, but we have to detect only vehicles that stop. To handle this situation, software is used so that, if an obstacle remains in place, the value generated is greater than the value generated when a vehicle simply passes in front of the IR sensor. Almost all long vehicles pass a point on a free road in less than five seconds. So, we set a time limit of 5000 ms, which can be changed according to requirements. If an object is detected by the IR sensor for more than five seconds (the threshold), it is deemed an accident.

### 5.3. Finding the Smoke Sensor Threshold

**Figure 12:**
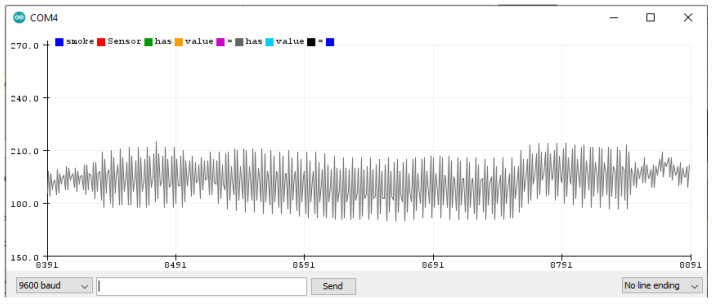
Smoke detector’s waveform when there is no smoke.

The smoke sensor has a normal range of about 150 to 220 when there is no smoke, as seen in Figure 12.

**Figure 13:**
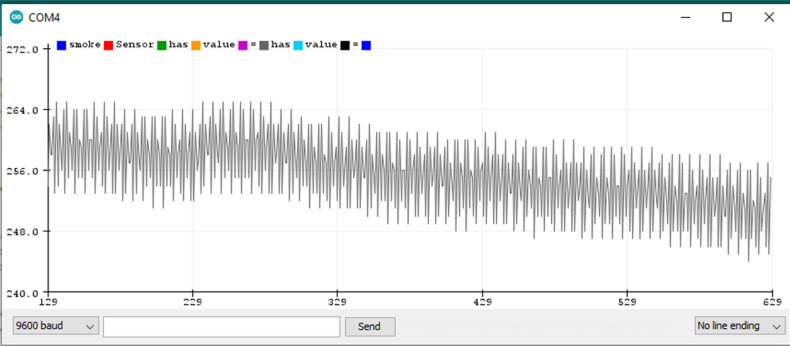
Smoke detector’s waveform when there is smoke.

Fire or smoke are found in the 230 to 270 sensor range, as seen in Figure 13. After performing several experiments, it is noticed that a value greater than 240 (the threshold) indicates fire.

### 5.4. Experiments for Finding Locations

**Figure 14:**
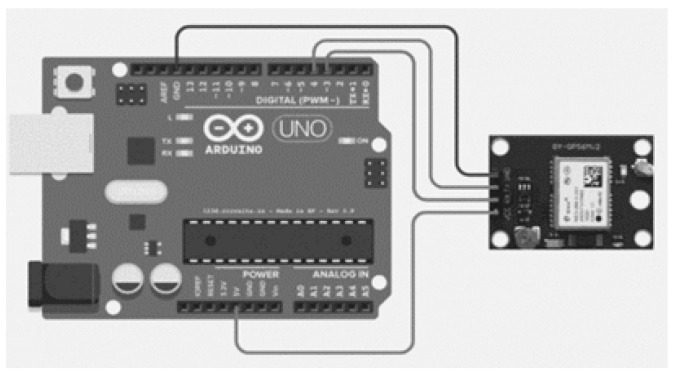
Wiring diagram of GPS 8M module connected to Arduino Uno board [69].

The GPS 8M module [69] is used for recording location information and saving it permanently. Because it takes longer to establish the first fix, once the location is determined that information is saved to the Arduino Uno board [70]. The temporary connection of the GPS 8M module with the Arduino Uno board is shown in Figure 14.

**Figure 15:**
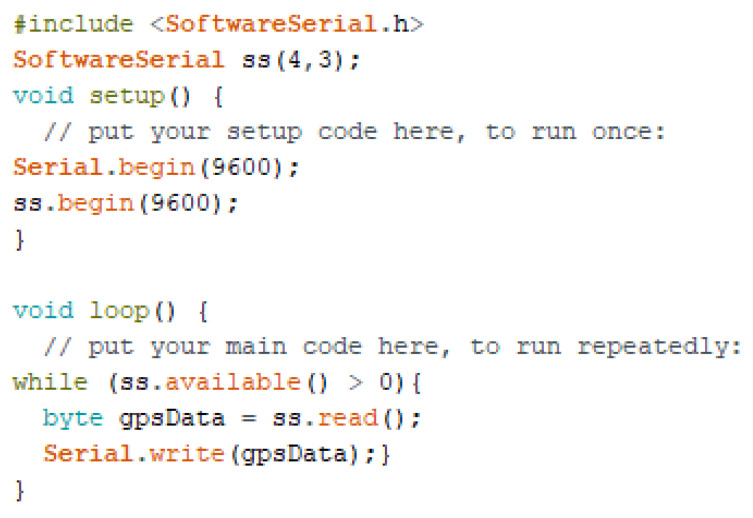
Sketch Uploaded to Arduino Uno Board.

A sketch is uploaded to the Arduino Uno board (as shown in Figure 15) to obtain the location information on the serial monitor.

When the GPS module is connected to the board, it sends the received data to a serial port. On the serial monitor, the data are viewed in the form of NMEA sentences. When it starts, the data received are not complete, as shown in the text box.

It takes (10 to 15 mins) to the module after a cold restart to obtain and lock in the data. The data are shown in another text box.

$GNVTG,,,,,,,,,N*2E

$GNGGA,,,,,,0,00,99.99,,,,,,*56

$GNGSA,A,1,,,,,,,,,,,,,99.99,99.99,99.99*2E

$GNGSA,A,1,,,,,,,,,,,,,99.99,99.99,99.99*2E

$GPGSV,1,1,00*79

$GLGSV,1,1,00*65

$GNGLL,,,,,,V,N*7A

$GNRMC,,V,,,,,,,,,,N*4D

From the numbers in the information shown on the serial monitor (below), we require only the highlighted information. It is copied and pasted to the freenmea.com website and parsed for the location. To obtain the exact latitude and longitude, the location is searched for by using Google Maps. The procedure or function is shown below.

$GNVTG,,T,,M,0.327,N,0.606,K,A*3B

$GNGGA,070234.00,3336.52621,N,07306.48790,E,1,09,1.02,514.3,M,−40.6,M,,*61

$GNGSA,A,3,24,15,17,28,19,02,,,,,,,1.72,1.02,1.39*14

$GNGSA,A,3,79,73,81,,,,,,,,,,1.72,1.02,1.39*13

$GPGSV,3,1,12,01,18,042,18,02,09,198,23,03,03,084,28,06,36,175,20*71

$GPGSV,3,2,12,15,12,271,25,17,68,012,25,19,71,278,27,21,00,034,*71

$GPGSV,3,3,12,22,01,058,,24,22,315,38,28,59,048,28,30,35,156,*7B

$GLGSV,2,1,07,73,14,322,34,78,01,131,,79,47,111,25,80,60,352,19*60

$GLGSV,2,2,07,81,28,030,26,82,81,025,,83,46,208,*51

$GNGLL,3336.52621,N,07306.48790,E,070234.00,A,A*72

$GNRMC,070235.00,A,3336.52625,N,07306.48784,E,0.382,,280421,,,A*6F

We stored the latitude, longitude, and address permanently (in code) to the Arduino Uno as a pre-saved location. After obtaining and saving the information, the GPS module was uninstalled and was no longer part of the system. The pre-saved location is used when an accident occurs. After the successful detection of an accident, the message on the location is sent to the immediately adjacent node.

The original NMEA sentence is:

$GNGLL,3336.52621,N,07306.48790,E,070234.00,A,A*72

The following output can be obtained from the execution of the above line:

33°36′31.33″N, 73°6′29.57″E

The following latitude and longitude values are shown when above coordinates are searched on the Google Maps:

33.608703, 73.108214

Finally, the following physical address is shown against the above latitude and longitude values:

“Street Number 7, Dhoke Raja Muhammad Khan, Rawalpindi, Islamabad Capital Territory 46000, Pakistan”

For the complete code for one node of the AALS, see Appendix B.

### 5.5. Validation of AALS

Each module is tested for proper working order and was found to operate as expected. All modules of the AALS system were integrated to build a complete working system. It works as a single unit where at least two nodes must be present at a distance of 50 m apart. Therefore, two nodes were built in a lab for testing and validation. By default, each node was in the listening position with all its sensing capabilities. Each node is able to detect accidents through the mic, IR sensor, and smoke sensor modules. Whenever an accident occurs in the area of a node, only that node responds and sends messages to another node. In this experimental setup, if a node has only one HC-12 communication module (i.e., to both receive and send a message), then it might suffer from the message loopback problem due to the echo of the same message. To overcome this problem, two HC-12 modules were used, tuned to different channels: one module for transforming and another for receiving. Each pair of nodes communicated with each other in an unidirectional manner. The transmitting module channel was the same as the receiving channel of the other module. The final AALS system node has two HC-12 wireless communication modules: one for receiving and the other for transmitting. After the experiments were completed, we made the following observations.

When there are 20 nodes along a 1 km stretch of road:

Minimum transmission time = 80 ms = 0.08 s;

Maximum transmission time = 1600 ms = 1.6 s.

When there are 2000 nodes on a 100 km stretch of road:

Minimum transmission time = 8000 ms = 8 s;

Maximum transmission time = 160,000 ms = 160 s.

The HC-12′s signal transmission time from one node to another node ranged between 4 ms and 80 ms.

1. When there was an accident, the time to detect it with sound was 100 ms.
2. The time to detect an accident using IR was 100 ms + Threshold (e.g., 100 + 5000 = 5100 ms).
3. The time required to detect smoke was 30 to 60 s.

The salient features of the AALS system node are as follows.

1. It can sense the sound produced by an accident/crash.
2. It can sense smoke from a fire.
3. It can sense an obstacle on the road for a period longer than the set threshold, e.g., 10 s.
4. When an accident was detected by sound or obstacle detection, the alert comprising light and sound was generated on the node.
5. A message with the location, traffic direction, and fire detection information was sent to the immediately adjacent node.
6. When a message about an accident was received by a node, it retransmitted the message to its adjacent node. This process then continued from node to node until the message was received by the EOC.
7. Each node that entered the retransmission phase also generated an alert using light and sound to warn oncoming vehicles.
8. All oncoming vehicles’ drivers and passengers were able to see the blinking lights and can hear the siren. An oncoming vehicle’s driver ensured their safety by taking precautionary measures.
9. When a rescue team reached the accident location and completed their tasks, the node was reset. All nodes were reset when they received the RESET message from the node that started the communication.

## 6. Conclusions and Future Work

The proposed AALS system provides a solution to the problems of actual location detection of an accident in order to protect other drivers and the authorities. It provides an alert, warning drivers of nEVs and EVs about an accident ahead. It helps the ambulance and fire brigade reach the destination easily by providing the exact location of the incident. Each node is a small but complete system that can sense its surroundings, and, in the case of an accident, generates alerts comprising a blinking light and a siren. The alerts will help drivers of oncoming vehicles to protect themselves from a mishap. Each node is responsible for communicating in one direction opposite the flow of traffic. By making smart roads using this AALS system, drivers are informed in time about any dangerous situation on the road. Moreover, in the case of an accident, the rescue team can reach the site without delay through an automatic process. Once the system is established, it works for a long time with limited maintenance. For better results, the EOCs should be at equidistant locations about 40–50 km apart at maximum. Each EOC should be equipped with an ambulance and fire brigade and should be responsible for sending a rescue team to the incident location. The EOC should also be aware of any ambulance and/or fire brigade already moving that might be near the location.

Current work is limited as the proposed system was installed and tested in a lab by simulating various accident scenarios. All results were found to be accurate after experiments. However, differences between the lab environment and the actual road environment may cause test results to differ from those expected in the operational environment. We will investigate the detection accuracy of the proposed system in a more practical situation on the road in our future work. An enhanced version of the AALS can be developed that will detect an accident from a longer distance (more than 100 km). Automatic fire extinguishers can be used to extinguish fires without the need to send a fire brigade. Other sensors can be used to provide information about the road conditions to drivers of nEVs and EVs through different display mechanisms. The same procedure can be applied to an individual lane to show its status (e.g., traffic density, single-lane incidents).

## Data Availability

Not applicable.

## Appendix A. Analysis of Previous Approaches

Table A1 provides a summary of the previous research with their adopted techniques and limitations.

**Table A1:** State-of-the-art accident avoidance approaches.

**Ref.**	**Technique/Technology**	**Limitations**
[5]	Ultrasonic sensors and PIC controller	Pre-accident and specific to EVs
[6]	Camera, image processing	High computational costs; pre-accident and EVs only
[7]	NDT, semi-Markov process	The problem of localization can be solved in a limited small area
[8]	Spatial data radar sensor	High computational costs Small errors between consecutive sensor readings will accumulate into large errors when summed over a long time [14]
[10]	Cruise control technology, PID, APS	It is not completely autonomous It may generate inaccurate results during bad weather (fog, rain) or driving through tunnels
[11]	DL	Limited to automobile crashes only, not motorbikes, bicycles, and pedestrians Infeasible results due to poor illumination
[12]	LiDAR, stop sight distance algorithm	Considered a fixed weight for vehicles
[13]	3D LiDAR, clustering, Kalman filter	Difficult to discern between neighboring objects; low tracking resilience for objects moving quickly
[14]	LiDAR, clustering, and classification	If two targets occupy neighboring segments, or share the same segment where longitudinal separation is less than 0.8 m, the proposed clustering algorithm can group them into a single cluster, resulting in an unresolved measurement
[15]	LiDAR, convex hull algorithm	Accuracy of contour prediction may be damaged due to higher degree curve and noise-induced change in reflection points
[17]	Onboard sensors, roadside units, GPS, Bluetooth, Ethernet	Specific to EVs Internet connectivity required
[19]	Heuristic technique, V2V, and V2I	They did not consider the environment of human-driven vehicles and lane-changing behavior in different and unexplored places
[20]	Maximum likelihood estimation, sensors, GPS, V2V	High deployment costs; can handle only a linear function
[21]	DL, convolution network-CNVPS (GCN-CNVPS), GPS, V2V	Assumptions made for GPS errors
[22]	Fuzzy logic, Dempster-Shafer Theory	Communication issues not discussed
[23]	Collision avoidance algorithm	Collisions can be identified and avoided in connected vehicles only
[24]	CCTV surveillance footage	Poor results for small vehicles The accident process may take longer due to heuristics
[25]	Cellular network, multi-hop ideal sending calculation	Probability of failure due to unpredicted behaviors in traffic
[26]	V2X	Cellular users are given first priority, followed by vehicular users
[27]	LTE	Scalability and suboptimal-channel issues
[28]	ITS-G5, LTE-V2X	Physical layer structure; synchronization problems in LTE-V2X [50]
[30]	GPS, smartphone sensors, and ZigBee	GPS-related problems Prone to false positives
[31]	Wave sensors, GPS, ZigBee	ABS technology GPS problems; specific to EVs only
[32]	Pulse-based short-range radar, GPS	The approach cannot handle unexpected objects in dynamic surroundings RMS errors are 7.3 cm laterally and 37.7 cm longitudinally; worst case: 27.8 cm and 115.1 cm, respectively [33]
[35,36]	CCTV and a Calogero–Moser system	May generate inaccurate results in bad weather
[37]	LiDAR, camera	The production of a real-time map of the environment from a sparse 3D point cloud is a key shortcoming of LiDAR
[38]	VANETs and cellular technology, biomedical sensors	High computational costs, big-data handling, specific to EVs only
[39]	Accelerometer, GSM, GPS, Arduino	EV-specific; no communication with other vehicles GPS-related problems
[40]	GSM, GPS-based	GPS initialization problem, EVs only
[41]	Smartphone-based	The fundamental issue is that it relies on a web server for notifications. There is no system in place that allows individual responders to track the location of an accident
[42]	Smartphone-based, wireless communication	Prone to false positives
[43]	GSM, GPRS, GPS	High costs GPS-related problems
[44]	LTE, 5G	-
[45]	V2X, 802.11bd and 5G NR, Edge Computing	-
[46]	VVLN, FD	-
[47]	WSN	Information about road conditions is provided to drivers
[48]	DSRC	Issues arises due to low car density, especially if there is no car or single car
[49]	RL	Focus of the work is to reduce traffic congestion by controlling traffic lights
[50]	DRL	Communication resources are assigned to improve system capacity

## Appendix B. Code of the AALS System for One Node

//This code is built for “Autonomous Accident Detection & Alert for Non-equipped Vehicles”

// This code is the property of Muhammad Zahid Hanif, MSCS FUUAST ISB.

// Library added for code simplification

#include <**SoftwareSerial**.h> // For serial communication though digital pins

#include <LCD_I2C.h>

LCD_I2C lcd(0x27); // Default address

**SoftwareSerial** HC12(2, 5); // HC-12 TX Pin, HC-12 RX Pin

// Variable Declarations

// Pin mapping

const int lightPin = 13; // used for alert by blinking light

const int sirenPin = 12; // used for alert by siren sound

const int resetPin = 11; // used for reset with pushbutton

const int softresetPin = 10; // software-based reset

const int irSensor = A0; // used for IR sensor input

const int micSensor = A1; // used for mic sensor input

const int smokeSensor = A2; // used for smoke sensor input

//PRE- Location Save

const String GPSLatitude = “32.783280”;

const String GPSLongitude = “72.703564”;

const String mylocation = “Lat:32.783280, Long:72.703564; Kallar Kahar, Chakwal, Punjab, Pakistan, N2S”;

// Sensor threshold settings

const int irThreshold = 100;// less than this threshold detects obstacle

const int micThreshold = 540; // greater than this threshold detects accident

const int smokeThreshold = 240; // greater than this value indicates fire

const int timeElapsedThreshold = 5000; // 5 s is for a long vehicle passing by

// Variables for storage input data

int irSensorValue = 0;

int micSensorValue = 0;

int smokeSensorValue = 0;

int resetPinValue = 0;

int timeElapse = 0;

int accidentSound = 0;

int fireDetected = 0;

void setup () {

// run once:

**Serial**.begin(9600); // serial baud rate is set to 9600

HC12.begin(9600); // Serial port to HC12

// gps.begin(9600); // Serial port to GPS

lcd.begin(); // If using more I2C devices using the wire library use lcd.begin(false)

lcd.backlight();

pinMode (lightPin, OUTPUT); // Declare lightPin as output pin

pinMode (sirenPin, OUTPUT); // Declare sirenPin as output pin

pinMode (resetPin, INPUT); // Declare sirenPin as input pin

pinMode (irSensor, INPUT); // Declare irSensor as input pin

pinMode (micSensor, INPUT); // Declare micSensor as input pin

pinMode (smokeSensor, INPUT);// Declare smokeSensor as input pin

pinMode (softresetPin, OUTPUT); // Connect this pin to reset of Arduino Uno board

digitalWrite (softresetPin, HIGH); // Make it high because reset is low

}

void loop() {

// runs repeatedly:

// get values from input pins

irSensorValue = analogRead (irSensor);

micSensorValue = analogRead (micSensor);

smokeSensorValue = analogRead (smokeSensor);

resetPinValue = digitalRead (resetPin);

// Check if hardreset button is pushed

if (resetPinValue == 1) {

HC12.write(“Reset”);

**Serial**.println(“HardReset”);

digitalWrite (softresetPin, LOW);

}

// serial print the values of sensors

**Serial**.print(“IR Sensor has value = “);

**Serial**.println(irSensorValue);

**Serial**.print(“mic Sensor has value = “);

**Serial**.println(micSensorValue);

**Serial**.print(“smoke Sensor has value = “);

**Serial**.println(smokeSensorValue);

// comparision of sensor values

if (irSensorValue < irThreshold) {

timeElapse += 100;

**Serial**.println(timeElapse);

}

if ((timeElapse <= timeElapsedThreshold) && (irSensorValue > irThreshold)) {

timeElapse = 0;

}

if (micSensorValue > micThreshold) {

accidentSound = 1;

**Serial**.println(“Accident sound detected”);

}

if (smokeSensorValue > smokeThreshold) {

fireDetected = 1;

**Serial**.println(“Fire detected”);

}

// Send message if accident detected

if ((accidentSound == 1) || (timeElapse >= timeElapsedThreshhold)) {

HC12.write(“Lat:32.783280,Long:72.703564;Kallar Kahar,Chakwal, Punjab, Pakistan, N2S”);

**Serial**.println(“Accident Detected”);

HC12.write(“Accident Detected”);

tone(sirenPin, 1000, 70);

digitalWrite(lightPin, HIGH);

if (fireDetected == 1) {

HC12.write(“Help: Fire brigade “);

**Serial**.println(“Help: Fire brigade “);

}

}

// lcd display

lcd.print (“Lati: “);

lcd.print (GPSLatitude); // print smoke detector value

lcd.setCursor(0, 1); // curser position

lcd.print(“long: “);

lcd.print(GPSLongitude);// print IR sensor value

lcd.backlight();

// if msg received by HC12 module

// HC12 Serial communication using pin 2 and pin 5

String MsgReceive = ““;

byte incomingByte;

while (HC12.available() > 0) {

incomingByte = HC12.read();

//HC12.write(incomingByte);

MsgReceive += char(incomingByte); // incoming msg is stored in MsgReceive variable

// changing

timeElapse = 5500;

// Serial.println(“zahid while”); //////

tone(sirenPin, 1000, 70);

digitalWrite(lightPin, HIGH);

// Serial.write(HC12.read()); // Send data to serial monitor //////

}

**Serial**.println(MsgReceive);

if (MsgReceive == ““) {

//Serial.println(“No Message Receive”); ;

}

else if (MsgReceive == “Reset”)

{

digitalWrite(lightPin, LOW);

digitalWrite(sirenPin, LOW);

irSensorValue = 0;

micSensorValue = 0;

smokeSensorValue = 0;

resetPinValue = 0;

timeElapse = 0;

accidentSound = 0;

fireDetected = 0;

}

else if (MsgReceive != ““)

{ tone(sirenPin, 1000, 70);

digitalWrite(lightPin, HIGH);

}

delay(100); // Wait for 50 milliseconds

lcd.clear();

//Serial.println(timeElapse);

}
